# Effect of pregnancy weight gain on infant birth weight among mothers attending antenatal care from private clinics in Mekelle City, Northern Ethiopia: A facility based follow-up study

**DOI:** 10.1371/journal.pone.0212424

**Published:** 2019-03-11

**Authors:** Freweini Gebrearegay Tela, Afework Mulugeta Bezabih, Amaha Kahsay Adhanu

**Affiliations:** Department of Nutrition and Dietetics, School of Public Health, College of Health Sciences, Mekelle University, Tigray, Ethiopia; Tulane University School of Public Health and Tropical Medicine, UNITED STATES

## Abstract

**Introduction:**

Weight gain during pregnancy is an important indicator of maternal and fetal nutrition during pregnancy. However, information regarding the effect of pregnancy weight gain on birth weight is lacking from developing countries.

**Objective:**

To determine the effect of pregnancy weight gain on the newborn’s birth weight in mothers attending antenatal care (ANC) services from private clinics.

**Methods:**

Health facility-based follow-up study was conducted among 332 pregnant mothers attending their antenatal care in Mekelle city, from October 2016 to June 2017. Before 28 weeks of gestation, pregnancy weight was collected retrospectively, then, mothers were followed-up until the time of infant delivery to record their birth weight. Data were also collected by a structured questionnaire and checklists and analyzed using SPSS version 21. The relationship between dependent and independent variables was assessed and presented using descriptive statistics, as well as t-test, ANOVA, and multivariable linear regression analysis. Variables:—pre-pregnancy BMI, maternal age, parity, decision making power on monetary resources, pregnancy interval, availability of housemaid, women dietary diversity score, maternal occupation, and pregnancy weight gain were included in the multivariable analysis.

**Results:**

Maternal weight increased monthly at a mean ± SD rate of 2 ± 0.7 kg in the second trimester, and 1.5 ± 0.7 kg in the third trimester. The mean ± SD of pre-pregnancy body mass index (BMI) and total pregnancy weight gain was 23.8 ± 4.6 kg/m^2^, and 12 ± 2.8 kg respectively. The mean ± SD of birth weight was 3440 ± 542 grams. Weight gain has a significant effect on infant birth weight, a 1 kg increase in the pregnancy weight was associated with 94 g increase in BW (β = 97, 95% CI: 73–120). After dividing the pre-pregnancy weight into four groups (< 18.5, 18.5–24.9, 25–29.9 and ≥30) kg/m^2^ based on the Institute of Medicine (IOM), we found a statistically significant birth weight difference between each group.

**Conclusion:**

Pregnancy weight gain has a significant effect on birth weight. Thus, ANC counseling services should focus on maternal weight gain to prevent sub-optimal birth weight.

## Introduction

A woman’s weight normally increases during pregnancy because of the growth of fetal and maternal tissues and fluids [1]. Weight gain during pregnancy, among the important indicators of pregnancy maternal nutrition, is also a good measure of intra-uterine fetal nutrition [2, 3]. Thus, sub-optimal gestational weight gain (GWG) is associated with various adverse pregnancy outcomes. These outcomes may include, but are not limited to: high birth weight (HBW), low birth weight (LBW), pregnancy-induced hypertension, gestational diabetes, preterm births, caesarean delivery, and delayed initiation of breastfeeding [3–5].

Birth weight (BW) is also a known predictor of fetal wellbeing and newborn's future chances of survival and is dependent on maternal health and nutrition during pregnancy [6]. It is also accepted that child growth failure occurs in the critical window of opportunity, from conception up-to two years of age, and about 50% of the growth failure which occurs by two years of age occurs in uterus [7]. Though some catch-up growth can occur among infants born with LBW, they never catch up in the same way as normal birth weight (NBW) babies [7]. Given its prediction of fetal wellbeing, intrauterine malnutrition has more serious and far reaching consequences [8], because an insult which occurs during pregnancy permanently affects tissue structure and function. These concerns make the fetal period a critical window of opportunity, and nutrition intervention during this period and improving BW will help break the vicious intergenerational cycle of malnutrition [7].

Despite exiting proven interventions during pregnancy including: weight monitoring, health education, and counseling on weight management, nutrition and physical activity, the majority of pregnant mothers in developing countries do not have access to this information and these services [9]. Thus, the issue of pregnancy weight gain remained of low priority to ANC providers and pregnant mothers from developing countries [10].

In 2009, the Institute of Medicine (IOM) has published a weight gain guideline for singleton pregnancy based on pre-pregnancy body mass index (BMI) classes. That is, for underweight mothers, a recommended GWG of: 12.5–18 kg, for normal weight: 11.5–16 kg, for overweight: 7–11.5 kg, and for obese mothers: 5–9 kg [9].

Pregnancy weight gain, irrespective of pre-pregnancy body mass index (BMI), is an independent predictor and found to have a significant effect on fetal growth [1, 6]. However, most of the previous studies were conducted in developed countries, and information regarding the effect of pregnancy weight gain on birth weight is lacking from developing countries [1, 9]. This study was therefore, aimed at determining the effect of pregnancy weight gain on birth weight among pregnant mothers getting antenatal care from private clinics in Mekelle city, Northern Ethiopia.

## Methods and materials

### Study area, study period and study population

The study was conducted from October 2016 to June 2017 among pregnant women attending ANC in selected private health facilities of Mekelle city, Tigray, which is one of the nine regions in Ethiopia. Mekelle is the capital city of Tigray region and is located 780 kilometers north of Addis Ababa, the capital city of the country. It has seven sub-cities and 33 Kebelles. It has one comprehensive specialized hospital, two general hospitals, and nine health centers. Mekelle city has a total population of 358,529 and 12,333 estimated pregnant mothers by the year 2016/17 which is projected from 2014 Ethiopian Central Statistical Agency (ECSA).

### Study design and setting

A prospective, health facility-based follow-up study design was employed.

### Inclusion and exclusion criteria

Women who had singleton pregnancies were included in this study. Whereas, women with hearing and speaking difficulty, with pre-existing or current medical conditions, and women with twin pregnancy were excluded from the study.

### Sample size calculation

The sample size was calculated using Open-epi version-2.3 with single population proportion formula with the assumption of 95% CI and 0.05 margin of error. Taking P = 28% from a study [11], the total sample size became 302. Adding a 10% non-response rate, the final sample size became 332.

### Sampling technique

Five clinics were selected based on their caseload. Using the flow of pregnant women who visit ANC services in the selected clinics in the same months of the previous year as a baseline, the number of study participants were assigned proportionally to each clinic. Mathematically, the average number of pregnant women who attended antenatal care in each clinic per month was multiplied by the total sample size (n = 332), divided by the total number of pregnant women who attended in the five clinics per month (391). All mothers coming to these clinics during the study period were enrolled consecutively until the sample size was attained.

### Methods of data collection

Pregnancy weight was collected retrospectively and prospectively. Before 28 weeks of gestation, monthly weight was collected retrospectively from mother’s ANC card and then, mothers were followed-up until the time of delivery to record the BW. The weight of mothers was measured while they were attending ANC services. BW was collected from the institution where each mother delivered her baby. The institutions where the mothers delivered were traced through phone contacts with the mothers. The height of the mother was measured on barefoot using height measuring board in a standing position and recorded to the nearest 0.1 centimeters. Maternal weight was measured using digital weight measuring scale (in three clinics), and Seca scale (in two clinics), and was recorded accurately to 0.1 kg. Data were also collected by an interviewer-administered structured questionnaire.

Women Dietary Diversity Scores (WDDS) were calculated from a single 24-hour dietary recall. All foods and liquids that were consumed a day before the data collection were categorized into 9 food groups namely: starchy staples, dark green leafy vegetables, vitamin A rich fruits/vegetables, other fruits/vegetables, flesh meat, organ meat, egg, legumes, and milk. A score of 1 was assigned to those who consumed a food item from any of the groups and if no food was taken a score of 0 was given. Accordingly, a score of up to 9 points was computed by summing up the values of all the groups, and it was classified as low (≤3), medium (4–6) and high (7–9) [12].

Mothers were asked their ability to decide on monetary resources especially for nutrition services at home, and it was asked if they receive money from their husband or they own it by themselves.

Data was collected by the trained midwives of the clinics and crosschecked by the principal investigator to ensure completeness.

### Data quality assurance

Two days training was given for data collectors and supervisors to have a common understanding of the overall methodology of the study to reduce systematic error. Before the actual data collection, the questionnaire was pre-tested, and appropriate modification was made based on the results of the pretest. The questionnaire was translated into local language, Tigrigna, and then translated back to English to ensure consistency of concepts. The maternal weight measuring scale was standardized and calibrated after measuring each study subjects. First-year nutrition graduate students were recruited as supervisors and checked the data collection process, accuracy and completeness periodically. The collected data was checked and cleaned before and after data entry.

### Data analysis

After the questionnaire was coded, data were entered using EPI data—3.1 and exported to SPSS—21 for analysis. It was cleaned by sorting and tabulating simple frequency tables. Total weight gain was determined as the difference between the weight of each woman at the last visit (at around a ninth month) and weight recorded at the first visit (at around 12 weeks). Variables were described using frequency and mean ± standard deviation. The relationship between BW and maternal weight gain were assessed using a linear model.

The pre-requisites namely linearity, independence, normality and homoscedasticity were checked before we ran the model. Shapiro Wilk's test (p > 0.05) showed that BW was approximately normally distributed for the predictor variables. Durbin-Watson < 4, and scatter plots also showed that observations are independent and linear respectively. Multicollinearity was tested using variance inflation factor (VIF) which was less than five for all of the independent variables.

Analysis of variance (ANOVA) and independent t-test was applied for testing mean BW differences in the different variables. For the variables which were found to be significant in ANOVA, we applied post-hoc (multiple comparisons) test to identify which pair of means were significantly different. Multivariable linear regression was also performed to determine the independent effect of gestational weight gain on birth weight, adjusting for other factors (pre-pregnancy BMI, maternal age, parity, inter-pregnancy interval, decision making on monetary resources, and women dietary diversity score.

To investigate the independent effect of GWG on BW, two sets of independent variables were entered in steps using the simultaneous regression (enter method) with variables retained in sequential blocks. The first block included pre-pregnancy BMI, maternal age, parity, inter-pregnancy interval, decision making on monetary resources, and women dietary diversity score. The second block included total pregnancy weight gain and all the variables that were entered in the first block. The outcome variable was birth weight.

Adjusted R^2^ was taken to determine the proportion of variation that was explained by the regression model. P-value < 0.05 and 95% CI were used to assess statistical significance.

### Ethical consideration

The study was conducted after ethical approval from the institutional review board (IRB) of the College of Health Sciences of Mekelle University was obtained. An official letter was taken from the School of Public Health to the responsible body. Permission from Regional Health Bureau, Mekelle Zone health office and the private health institutions was also sought. Verbal consent was taken from the participants after they were informed about the purposes and objectives of the study. Confidentiality and privacy were maintained throughout the study. The right of the participant to participate, refuse or stop at any time during the data collection process was guaranteed. The procedure constituted minimal risk to participants and this was explained before the start of the data collection or interviewing.

## Results

### Description of the respondents

Three hundred thirty two (332) pregnant women were recruited in this study, with a 93% response rate. Three hundred twenty eight (98.8%) of the mothers were from Tigray Region, and all of them were urban residents. Mean age of the mothers was 28.5 years, 198 (59.6%) of them being <30 years old (Table 1).

**Table 1:** Socio-demographic characteristics of pregnant women attending ANC of private clinics in Mekelle City, Tigray, Ethiopia, 2017, (n = 332).

Variable		Frequency (%)	Mean ± SD
Religion			
	Orthodox	283 (85.3)	
	Muslim	39 (14.7)	
	Others (catholic and protestant)	10 (3)	
Marital status			
	Single	46 (13.9)	
	Married	282 (84.9)	
	Others (widowed and divorced)	4 (1.2)	
Educational level			
	Primary education	21 (6.3)	
	Secondary education	111 (33.4)	
	Diploma and above	200 (60.2)	
Occupational status of the mother			
	Student	16 (4.8)	
	Housewife	112 (33.7)	
	Government employee	78 (23.5)	
	Non-governmental employee	45 (13.6)	
	Self–employed	81 (24.4)	
Ownership of monetary resources			
	Mostly father	130 (39.2)	
	Mostly mother	23 (6.9)	
	Both jointly	179 (53.9)	
Family size			
	< 4	179 (53.9)	4 ± 1
	≥ 4	153 (46.1)	
Availability of housemaid			
	No	115 (34.6)	
	Yes	217 (65.4)	

All of the mothers had attended ANC at least six times during their last two trimesters. Three hundred five (91.9%) of the pregnancies were planned and wanted. Three hundred three (98%) of the newborns were born at term. The time gap between the current and last pregnancy was less than three years for 39.6% of mothers.

The mean ± SD pre-pregnancy BMI was 23.8 ± 4.6 kg/m^2^. Mean weight gain in the 2^nd^ and the 3^rd^ trimesters of pregnancy were 6 ± 2 kg and 5 ± 2 kg respectively, and the mean weight gain in the entire pregnancy was 12 ± 3 kg. Maternal weight increased monthly at a mean ± SD rate of 2 ± 0.7 kg in the second trimester, and 1.5 ± 0.7 kg in the third trimester. WDDs varied from 1 to 9, with 68% having medium dietary diversity score, and 91 (27.4%) had high dietary diversity score.

### Mean birth weight difference of various maternal characteristics

In the ANOVA and t-test:- pre-pregnancy BMI, weight gain during pregnancy, parity availability of housemaid, interpregnancy interval, money for living activities, the age of the mother, and WDDs were significantly associated with different mean birth weight.

In the post-hoc (multiple comparisons) test, underweight women had significantly lighter babies as compared to overweight and obese women. But there was no significant BW difference between underweight and normal weight women. The obese women also gave significantly higher BW babies comparing with underweight and normal weight women. All of the households whose monetary resources were controlled jointly by both mother and father had significantly heavier babies when compared to HHs that are owned by father alone. But there was no significant mean BW difference between HHs owned by mother alone and HHs owned by both mother and father.

Primiparous women also gave birth to significantly lighter babies than multiparous mothers. But there was no significant mean BW difference between primiparous and grand multiparous (women who give birth five times or more) mothers. Mothers with low micronutrient adherence had lighter babies as compared to high and medium adherent mothers. But medium and high adherents had no significant BW difference.

Mean BW was also different among mothers with low and high dietary diversity score (WDDs). Mothers with high WDDs had significantly larger babies than those with the low score. But there was no significant mean BW difference between low and medium, as well as medium and high WDDS (Table 2).

**Table 2:** Mean BW difference between the various maternal and newborn characteristics among mothers attending ANC visit from private clinics, in Mekelle city, 2016.

	Characteristics	Mean ± SD	t/F	P-value	95% CI
Pre-pregnancy BMI(kg/m^2^)					
	< 18.5	3139 ± 471	8.385	< 0.0001	(2957, 3322)
	18.5–24.9	3379 ± 494			(3305, 3453)
	25–29.9	3576 ± 559			(3449, 3704)
	≥30	3709 ± 630			(3379, 3501)
Pregnancy interval					
	< 3 years	3278 ± 504	-5.611	<0.0001	(-544, -261)
	≥ 3 years	3676 ± 508			(-543, -261)
WDDS					
	Low WDDS	3150 ± 535			(2900, 3400)
	Medium WDDS	3342 ± 495	21.907	< 0.0001	(3273, 3410)
	High WDDS	3740 ± 535			(3625, 3854)
Child sex of the current pregnancy					
	Male	3451 ± 515	0.513	0.608	(-90, 153)
	Female	3420 ± 567			(-91, 154)
Occupation of the mother					
	Student	3253 ± 566	2.338	0.065	(2940, 3567)
	Government employee	3396 ± 517			(3278, 3514)
	Non-governmental employee	3583 ± 569			(3391, 3776)
	Self-employed	3542 ± 543			(3420,3663)
	Housewife	3372 ± 534			(3379, 3500)
Educational status of the mother					
	Primary education	3335 ± 681	0.416	0.66	(3016, 3654)
	Secondary education	3440 ± 540			(3334, 3545)
	Diploma and above	3452 ± 529			(3375, 3528)
Availability of housemaid					
	No	3290 ± 574	-3.567	0.044	(-354, -102)
	Yes	3519 ± 510			(-359, -97)

### The independent effect of gestational weight gain on birth weight

The first block of the regression model which included: pre-pregnancy BMI, maternal age, maternal occupation, parity, pregnancy interval, availability of housemaid, and women dietary diversity score accounted for a statistically significant variation in BW (adjusted R^2^ = 23.4%, p <0.0001), and the second block which adds GWG to the first block also accounted for statistically significant variation in BW (adjusted R^2^ = 42.2%, p < 0.0001) implying that 42.2% of the total variation in BW was explained by the independent variables namely: GWG, pre-pregnancy BMI, and interpregnancy interval. Almost nineteen percent (18.8%) of this variation was contributed by pregnancy weight gain. A one kilogram increase in the pregnancy weight was associated with a 97g increase in BW, holding the other variables constant (Table 3).

**Table 3:** Multiple linear modeling for the associations between maternal factors and birth weight among mothers attending ANC visits from private clinics in Mekelle city, 2016/17 (309).

Characteristics	β (95% CI)	P-value
Pregnancy weight gain	97 (73, 120)	<0.0001
Pre-pregnancy BMI	0.369 (29, 54)	<0.0001
Inter–pregnancy interval ≥ 3 years	0.235 (142, 379)	<0.0001
Women dietary diversity	0.066 (- 13, 54)	0.23
Age of the mother	0.033 (- 10, 20)	0.54
Housemaid available	0.063 (-50, 299)	0.238

## Discussion

Considering the wider implementation of ANC in all public and many private health institutions in Ethiopia, a study regarding the effect of pregnancy weight gain on BW from such institutions is lacking. This study basically determined the effect of gestational weight gain on BW among mothers attending ANC from private clinics. Though direct comparison might be unreasonable, some findings of this study seemed to be comparable with some of the studies from developed countries [13–16].

Gestational weight gain was found to have a significant effect on birth weight. Based on this study, a 1 kg increase in pregnancy weight is associated with a 97-gram increase in BW. This might be due to the fact that, weight gain during pregnancy, which is an important indicator of nutrition in pregnancy, significantly contributes to intra-uterine fetal growth [2]. This was supported by a study in Taiwan which depicted that pregnancy weight gain was the highest contributor to the variation in BW [13]. This was also similar to a study in Iran, which showed that mean of GWG of women who gave birth to an infant with a BW of <2500g was significantly less than the mean GWG of women who gave a BW of > 2500 g [3].

Literature from Thailand and China also stated that the mean BW in mothers with higher GWG was significantly greater than the mean BW in those with a lower weight gain [14, 15]. Similarly, a within family analysis in US suggested that excess weight gain significantly increases the risk of having HBW babies, whereas, inadequate GWG increases the incidence of LBW [17]. A study by Sommer and colleagues also revealed that GWG is the strongest independent predictor of BW with a 0.21 kg/week giving rise to 110.7 g heavier babies [17].

The study had some limitation including the chance of residual confounding even with controlling of potential confounding variables. There could be possible association between birth weight and other factors such as physiological, psychological, socio-cultural, and environmental factors [18].

To conclude, pregnancy weight gain has a significant effect on birth weight, and a 1kg increase in pregnancy weight was associated with a 97 g increase in birth weight. Therefore, pregnancy weight management should be actively promoted through intensive counseling during the routine ANC contacts.

## Supporting information

S1 File Questionnaire used for data collection. (PDF)
