# Supplementary material for: Effect of pregnancy weight gain on infant birth weight among mothers attending antenatal care from private clinics in Mekelle City, Northern Ethiopia: A facility based follow-up study
Source: PLoS One. 2019 Mar 11;14(3):e0212424. doi: 10.1371/journal.pone.0212424 (PMC6411121; doi:10.1371/journal.pone.0212424)
Supplement: S1 File — (PDF) [file pone.0212424.s001.pdf]

Questionnaire used for data collection

Part I: Questions on socio-demographic

S. No	Item	Response/ value
101	Age of the mother	-------------years
102	Ethnicity?	1.Tigray<br>2.Amara<br>3.Afar<br>4.Other(specify)______
103	Place of residence	1. Urban<br>2. Rural
104	Religion	1.Orthodox<br>2.Muslim<br>3.Catholic<br>4.protestant<br>5.Others
105	Current marital status	1.Single<br>2.Married<br>3. Divorced<br>4. Widowed<br>5.Other(specify)______
106	Educational level?	1.illitrate<br>2.primary education

		3.secondary education<br>4.diploma<br>5.degree and above
107	Current occupational status?	1.Student<br>2.Government employee<br>3. private employee<br>4.NGOs employee<br>5.Self-employed<br>6.House wife<br>7.day laborer<br>8.Seeking for job<br>9.Other (specify)_____
108	What is your family monthly income?	__________ETB
109	Total family members	__________in number
110	Did you have domestic worker?	1. No<br>Yes
111	Who is decision maker to use money for living activities?	1. mother<br>2. father<br>3.Both jointly<br>4.Others (specify)_____

Part II: Questions on Obstetric characteristics

201	How many live births do you have?	_______in number
202	Birth weight of your last pregnancy	________gram
203	What was the time gap between the previous and the current pregnancy?	___________years
204	What is the type the current pregnancy?	1. Unplanned and Unwanted<br>2. Unplanned but wanted<br>3. Planned and Wanted<br>4. I don't know

Part III: Questions on behavioral factors

301	Did you get any counseling service during Pregnancy?	0. No<br>1. Yes
302	What type of counseling did you get?	1. To follow ANC monthly<br>2. To take micronutrient tablets daily<br>3. Daily physical exercise<br>4. Nutrition counsling<br>5. On weight management<br>6. Monthly injection of TT vaccine<br>7. Birth preparedness<br>8. Others

303	Do you take additional food than usual?	0. No<br>1. Yes
304	How many times do you eat per day when non-pregnant?	1. Three times<br>2. Four times<br>3. more than 4 times
305	How many times do you eat per day during current pregnancy?	1. Three times<br>2. Four times<br>3. more than 4 times
306	Did you fast during your pregnancy?	No 2. Yes
307	Did you fast during your pregnancy?	No 2. Yes
308	If the answer for no 506 is yes; What do you fast?	1. Animal products only<br>2. Animal products and fast foods until 6 o'clock<br>Animal products and fast foods throughout the day
309	How is the status of your appetite (eating condition) during pregnancy?	1. Decreased<br>2. Increased<br>3. Not changed
310	Have you ever smoked during your current pregnancy?	1. No 2. Yes

311	Have you ever drunk alcoholics during your current pregnancy?	1. Yes 2. No
312	Physical exercise per day	1. Not at all<br>2. Less than 30 minutes<br>3. 30 minutes or more

Part IV: Questions on women dietary diversity

Did you eat any of the following foods (meal or snack) during the past 24 hours?

401	Did you eat any bread, biscuits, or any other foods made from cereal (maize, sorghum, millet, wheat, barely or Teff) or white potatoes, white cassava, or other foods made from roots yesterday?	0. No<br>1. Yes
402	Did you eat any vegetable such as pumpkin, carrot, yellow/orange flesh sweet potatoes, or any fruit like ripe mango, papaya, banana, avocado, lemon, orange or 100% juice made from them yesterday?	0. No<br>1. Yes
403	Did you eat any dark green leafy vegetables (kale, cassava leaves, Swiss chard, cabbage) yesterday?	0. No<br>1. Yes
404	Did you eat other vegetables (e.g. tomato, onion, or other locally available vegetables or other fruits, including wild fruits and 100% fruit juice made from these yesterday?	0. No<br>1. Yes
405	Did you eat any meat (beef, lamb, and goat, chicken) or fish and other seafood yesterday?	0. No<br>1. Yes

406	Did you eat any eggs yesterday?	0. No<br>1. Yes
407	Did you eat any organ meat (liver, kidney, and heart) yesterday?	0. No<br>1. Yes
408	Did you eat any food made from legumes like kidney beans, haricot beans, field peas, cow peas, chick peas, nuts, lentils or others?	0. No<br>1. Yes
409	Did you eat any milk, cheese, yogurt and other milk products yesterday?	0. No<br>1. Yes

Part V: Questions on Household Food Insecurity Access Scale (HFIAS)

For each of the following questions, consider what has happened in the past 30 days. If No, skip to the next question. If the answer is yes, how often did this happen?

-Rarely (once or twice) = 1

-Sometimes (3-10 times) = 2

-Often (more than 10 times) = 3

501	Did you worry that your household would not have enough food?	0. No<br>1. Yes	1.rarely<br>2.sometimes<br>3.often
502	Were you or any household member not able to eat the kinds of food you preferred because of lack of resources?	0. No<br>1. Yes	1.rarely<br>2.sometimes<br>3.often

503	Did you or any household member eat just a few kinds of food day after day due to lack of resources?	0. No<br>1. Yes	1.rarely<br>2.sometimes<br>3.often
504	Did you or any household member eat food that you preferred not to eat because of lack of resources to obtain other types of foods?	0. No<br>1. Yes	1.rarely<br>2.sometimes<br>3.often
505	Did you or any household member eat a smaller meal than you felt you needed because there was no enough food	0. No<br>1. Yes	1.rarely<br>2.sometimes<br>3.often
506	Did you or any household member eat fewer meals in a day because there was no enough food?	0. No<br>1. Yes	1.rarely<br>2.sometimes<br>3.often
507	Was there ever no food at all in your household because there were no enough resources to get more	0. No<br>1. Yes	1.rarely<br>2.sometimes<br>3.often
508	Did you or any household member go to sleep at night hungry because there was no enough food?	0. No<br>1. Yes	1.rarely<br>2.sometimes<br>3.often
509	Did you or any household member go a whole day without eating anything because there was no enough food?	0. No<br>1. Yes	1.rarely<br>2.sometimes<br>3.often

Part VI: Record review/checklist

601	pre-pregnancy weight of the mother	________________in Kg
602	Height of the mother	_______________in centimeters
603	Hemoglobin level at 1st ANC visit	_____________
604	Pregnancy weight	1. At 3rd month ________<br>2. At 4th month_________<br>3. At 5th month_________<br>4. At 6th month_________<br>5. At 7th month_________<br>6. At 8th month_________<br>7. At 9th month_________
605	Gestational age at delivery (weeks)	1. <37<br>2. 37-42<br>3. >42
606	Birth weight	__________gram
607	Sex of the newborn	1. Male<br>2. Female
